# Lapnurse—A Blended Learning Course for Nursing Education in Minimally Invasive Surgery: Design and Experts’ Preliminary Validation of Its Online Theoretical Module

**DOI:** 10.3390/healthcare9080951

**Published:** 2021-07-28

**Authors:** Juan Francisco Ortega-Morán, Blas Pagador, Juan Maestre-Antequera, Javier Sánchez-Fernández, Antonio Arco, Francisco Monteiro, Francisco M. Sánchez-Margallo

**Affiliations:** 1 Jesús Usón Minimally Invasive Surgery Centre, Ctra. N-521, Km. 41.8, 10071 Cáceres, Spain; jbpagador@ccmijesususon.com (B.P.); jmaestre@ccmijesususon.com (J.M.-A.); jsanchez@ccmijesususon.com (J.S.-F.); msanchez@ccmijesususon.com (F.M.S.-M.); 2 Polytechnic Institute of Portalegre, Praça do Município, 11, 7300-110 Portalegre, Portugal; a.arco@ipportalegre.pt (A.A.); franciscomonteiro@ipportalegre.pt (F.M.)

**Keywords:** blended learning, design, education, e-learning, laparoscopy, minimally invasive surgery, nursing, training course, validation

## Abstract

Background: The implantation of Minimally Invasive Surgery (MIS) leads to the specialization of nurses in this surgical field. However, there is no standard curriculum of MIS Nursing in Europe. Spanish and Portuguese nurses are inexperienced and have poor training in MIS. For that, a blended learning course for nursing education in MIS (Lapnurse) has been developed. This work aims to detail the course design and to preliminary validate by experts its online theoretical module. Methods: Lapnurse consists of an online module with nine theoretical lessons and a face-to-face module with three practical lessons. The e-learning environment created to provide the online module, with didactic contents based on surgical videos and innovative 3D designs, has been validated by two technicians (functionality) and four nurses with teaching experience in MIS (usability and content). Results: The E-learning platform meets all technical requirements, provides whole and updated multimedia contents correctly applied for educational purposes, incorporates interactivity with 3D designs, and has an attractive, easy-to-use and intuitive design. Conclusions: The lack of knowledge in MIS of Spanish and Portuguese nurses could be addressed by the blended learning course created, Lapnurse, where the e-learning environment that provides theoretical training has obtained a positive validation.

## 1. Introduction

Health professionals’ mobility between countries has been a common practice for many years. As an example of this, the migration of nurses between Spain and Portugal is easier because of their geographical contiguity and cultural affinity, and it has always existed in both directions [1]. That cross-border migration of nurses is enriching for both destination and origin countries, as long as these health professionals are adequately trained to practice in the destination country. Minimally Invasive Surgery (MIS) techniques have been implanted in recent years as a routine surgical practice, making it necessary to meet the training needs of professionals in this surgical field [2], including nurses. However, as far as we know, there is no standard curriculum of MIS Nursing in Europe. Thus, appropriate means should be available for nurses to acquire the knowledge that allows them to develop new attitudes and skills in the workplace [3], as required in MIS.

E-learning may be a good tool to improve the quality of education [4], allowing students flexibility in time, place, and access to content. Furthermore, it improves their learning ability by allowing them to progress at the most appropriate speed [5]. However, those education programs carried out entirely online can raise inequalities for users with computer illiteracy [6]. Moreover, e-learning has a series of interrelation drawbacks between teachers and students, such as the inability of students to receive immediate feedback from the teacher [5]. For that, blended learning, which combines both online and face-to-face learning, is a method that allows overcoming these barriers of e-learning, combining the flexibility of timing and the convenience of online delivery with the spontaneity and interpersonal interaction of face-to-face learning [7].

Although the benefits of e-learning and blended learning have been demonstrated in several studies, not enough is known about what nurses think about continuing education through e-learning [8,9] or blended learning [10]. For that, a study has been performed to firstly identify and compare training needs and level of experience in MIS of Spanish and Portuguese nurses, in order to check if they are qualified to work in this field, and to also check whether e-learning technologies would be useful for a successful MIS training. A total of 81 nurses from Extremadura (Spain) and 113 nurses from Alentejo (Portugal) participated by filling in a questionnaire that showed a lack of experience and training of nurses in MIS. In order to meet their training needs, they consider essential the implementation of e-learning environments that offer courses with useful contents for their work and a focus on clinical practice, to incorporate interactivity into these environments, and to use surgical videos in online training processes. Such study indicated that nurses are technologically competent to be trained by e-learning, and the blended learning is their preferred method to learn MIS.

Therefore, we have designed and developed a laparoscopic training course for nursing, named Lapnurse, through blended learning method with interactive didactic contents. The main objectives of this study were to:

- Detail how the course has been designed and developed based on the training needs demanded by nurses; and
- Preliminary assess by experts in a pilot study the functionality, content, and usability of the e-learning environment that provides the theoretical module of Lapnurse.

## 2. Materials and Methods

### 2.1. Course Design

**Figure 1:**
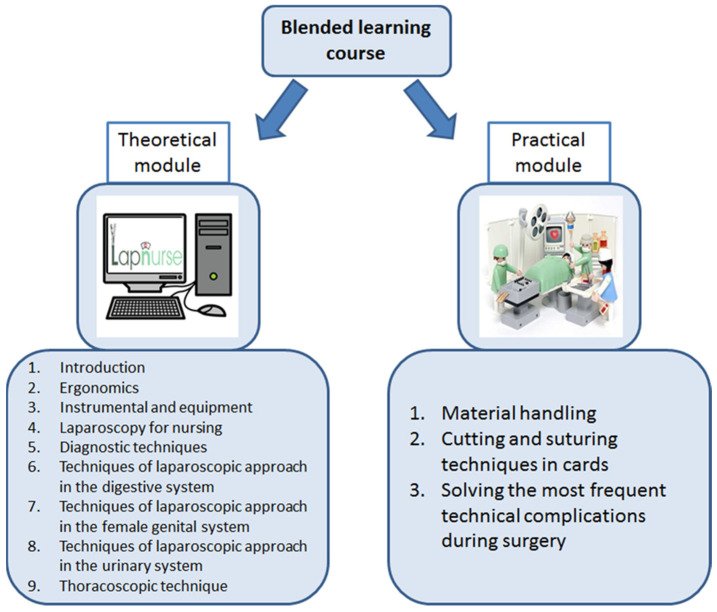
Blended learning course.

Blended learning method, as preferred by nurses, has been selected to implement the Lapnurse course. This course consists of (1) a theoretical module with nine lessons, to be taught through an e-learning web environment, and (2) a practical module with three lessons, to be carried out in person in a specialized reference centre (Figure 1).

The contents of the course have been designed based on the role of nurses in MIS. The tasks of circulating nurses are mainly focused on maintaining a safe and comfortable environment in the surgical room during the surgery. They do not participate in intervention and work outside of the sterile field, and have been responsible for the inspection of surgical equipment and instruments, and also the preparation of the documentation related to the surgery and the patient. However, the scrub nurses are directly involved in the surgical intervention working within the sterile field. They prepare the operating room for the patient and the instruments. They assist the surgical team by donning sterile masks, gloves, and gowns, as well as by passing the instruments during surgery. For this, surgical nurses need to acquire the necessary knowledge about instruments, equipment, and techniques related to laparoscopic interventions in order to perform their work in a safe way. Different sections of the developed course have been designed to meet all these training needs in MIS.

**Figure 2:**
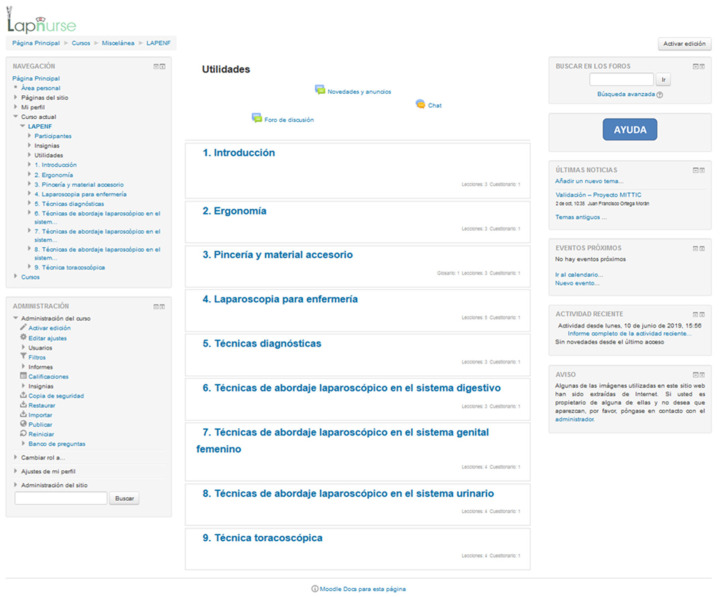
The Spanish version of the e-learning environment used to provide the theoretical module of Lapnurse.

As the e-learning environment used to provide the theoretical module of Lapnurse, the Moodle web application [11] has been selected (Figure 2). This free distribution course management system is very useful in the educational field, since it allows for incremental performance and satisfaction when compared to traditional didactic lectures [12].

Lessons consist of sequential pages providing all resources of the course (text, images, videos, and 3D designs), in Spanish and Portuguese languages. On the left side of the environment, the menu of each lesson is displayed, so that the user can view the tabs that the lesson contains and can navigate through them. At the end of each lesson, a questionnaire evaluates the knowledge acquired by the students through multiple choice and true/false questions, with two attempts to answer them within a limited time. Each attempt is automatically scored and the results are saved in the gradebook, although they are displayed to the user, with feedback comments and correct answers.

To encourage collaborative learning, synchronous and asynchronous means for communication between users have been included (chat and two forums, respectively). The chat allows participants to have a discussion in text format in a synchronous way in real time. Chats are especially useful when a group does not have the possibility to meet physically to talk face-to-face. The forum allows participants to have asynchronous discussions that take place over an extended period of time. The automatically created forum “News and announcement” indicates what is happening in the course, and it is only posted by teachers and an administrator. Additionally, the “Discussion forum” has been created, in which all users can add or reply to posts.

The main roles interacting with the system are the following:

- Student: They will be the main receiver of didactic contents in an active knowledge search. For aspects of formal and non-formal learning, the student acts as a consumer of content, e.g., accessing didactic content and courses, but without permission to create new content or edit existing ones. For aspects of informal learning, the student is considered an active participant within the professional network of the environment. They will participate in forums and discussions, and will interact with other users in the community.
- Teacher: A MIS surgeon with broad experience in teaching. As such, the main role of the teacher will be as a content provider for formal learning, creating and editing didactic contents, and loading them into the environment. For informal learning, they will be considered an active participant within the professional network of the environment. They will be able to upload multimedia content, participate as a tutor solving doubts in forums and debates, and interact with other users in the community.
- Administrator: The administrator will normally be an expert in computer systems, responsible for all management and technical aspects of the environment. Among their tasks are user management, system monitoring, and technical assistance.

### 2.2. Didactic Surgical Videos

Surgical videos are the basis of the didactic contents offered by Lapnurse, since they are very important and useful for nurses in online training processes and have increasingly been used for continuing medical education in e-learning technologies [13,14].

These are endoscopic videos corresponding to digestive, gynaecological, thoracic, and urologic surgical interventions. Video sections of interest to users have been selected from raw videos, avoiding long duration because the time away from work is the main drawback of health professionals who want training [15] and short contents are ideal for them [16]. A wide sample of up-to-date videos of each surgical specialty has been included to provide innovative information on advances in applied technology in line with the current surgical trend.

### 2.3. Interactive 3D Designs

**Figure 3:**
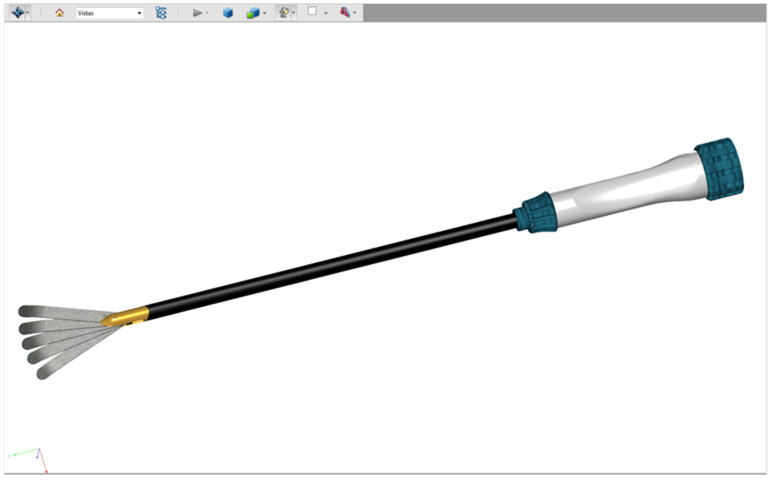
Interactive 3D design.

New interactive 3D designs of laparoscopic instrumental and equipment have been developed to incorporate the interactivity demanded by nurses. They are PDF files with the 3D model (Figure 3), which allow the user to perform some interactive actions over the objects, such as rotate and move the model, zoom in or out, generate defined views, add comments, make 3D measurements, make section views, and select visualization of each component of the design, among others.

All interactive 3D designs have been included in a Glossary within lesson 3. When any term of the glossary appears in the text of the course, that word is highlighted so that users can click it to visualize and interact with interactive 3D designs without having to visit the glossary.

### 2.4. Preliminary Validation by Experts of the Online Theoretical Module

**Figure 4:**
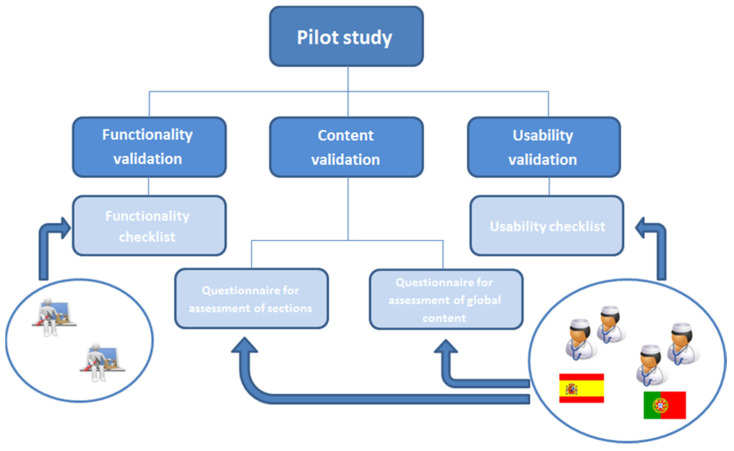
Scheme of the validation methodology.

A pilot study for a preliminary validation by experts of the online theoretical module of Lapnurse has been performed according to the scheme shown in Figure 4.

Functionality, contents, and usability tests have been performed in order to get an e-learning platform of high quality [17]. Functionality validation checks the correct functioning of the system, as well as compliance with the functional requirements, through a checklist (see Appendix A). Content validation evaluates the suitability of the lessons and contents offered to meet the learning requirements. For that, a five-point Likert scale survey (1—completely disagree, to 5—completely agree) has been used to subjectively evaluate the specific content of each lesson and the global content of the course (see Appendix B). The minimum threshold for considering a validation as positive has been set to 3.5 out of 5 points in the Likert scale, according to 70% of agreement [18]. A “yes/no” checklist with a list of requirements regarding the usability of the e-learning platform has been used to assess the design and layout of the web environment (see Appendix C).

Two technicians with extensive experience in the development of distance training tools (functionality validation) and four nurses (two from Spain and two from Portugal) with teaching experience and advanced knowledge in MIS (content and usability validation) carried out such tests. Participants had no relationship with the course or its creators, and the non-probability convenience sampling technique has been used to conduct sampling of participants once the course was developed, where emails were sent to contacts whose data are stored in databases of partners involved in the study. Four to five participants are enough for detecting usability problems in a preliminary validation of a web environment [19], and a questionnaire is the most usual method [20,21,22]. Results were not statistically analysed looking for significant differences between Spanish and Portuguese participants because it is not appropriate in groups of two users each. The analysis of the data was carried out using mean values, standard deviations, and percentages with the software SPSS 15.0 for Windows (IBM, Armonk, NY, USA).

## 3. Results

The two technical evaluators verified that the e-learning platform meets all technical requirements related to access management, user profile and interactions, environment maintenance, and formal and informal learning.

**Figure 5:**
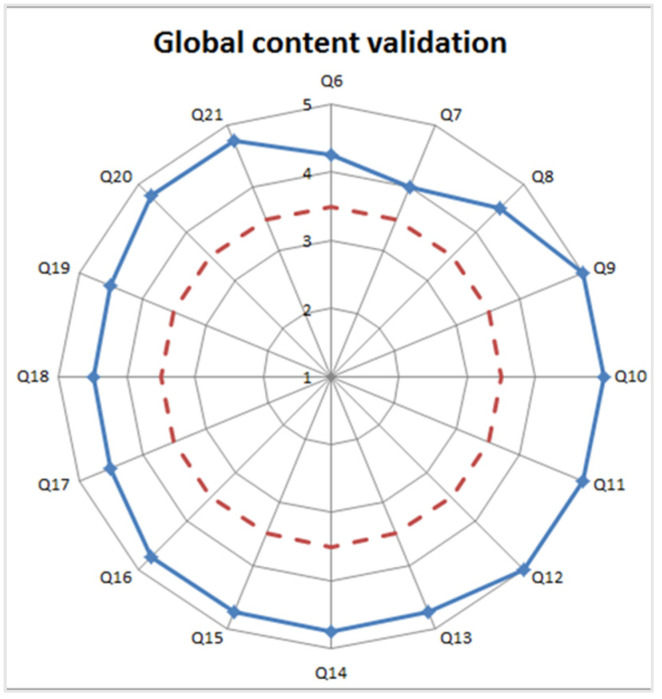
The blue continuous line represents the results of the validation of the global content of the course (1—Completely disagree, to 5—completely agree). The red dashed line indicates the minimum threshold necessary for positive validation.

Averaged results from the validation of specific contents of each lesson are shown in Table 1 and Table 2, and of the global contents in Figure 5. In both cases, all issues far exceed the validation threshold, most of them reaching almost the highest score.

Lessons most important for experts are instrumental and equipment, ergonomics, and laparoscopy for nursing, and the least interesting are those related to diagnostic and approach techniques. Resources most important are lessons and didactic contents, while the least interesting are the means of communications between users (chat and forum).

**Figure 6:**
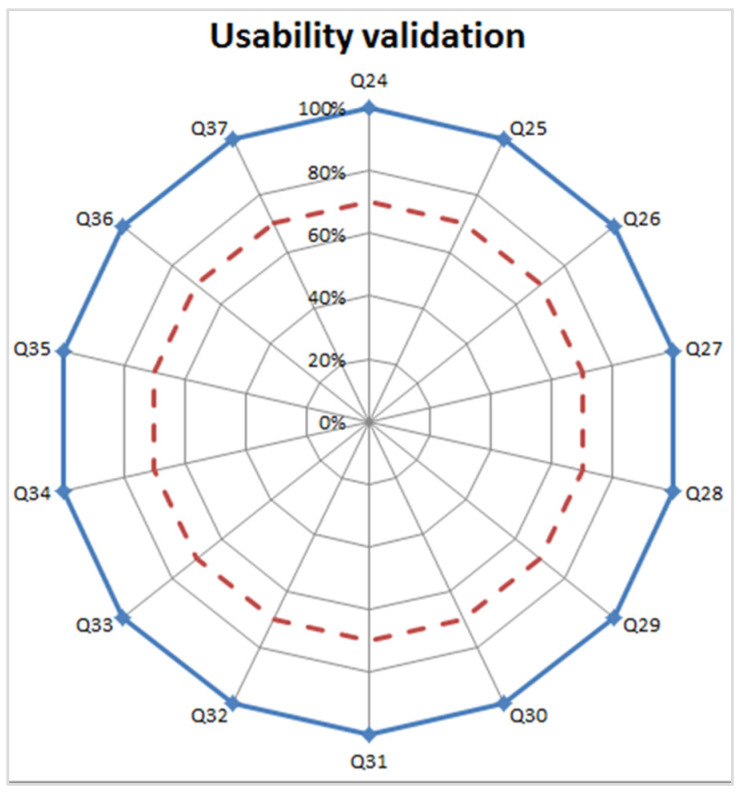
The blue continuous line represents the results of usability validation (% surgeons agree). The red dashed line indicates the minimum threshold necessary for positive validation.

Subjective usability results related to the design of the e-learning environment show a positive answer from all experts to all questions, as shown in Figure 6.

## 4. Discussion

The traditional used method for MIS training consisting of the attendance of nurses to in-person courses requires incorporating the technological multimedia innovation according to the growth and evolution of this emerging surgical technique. For that, the laparoscopic training course for nursing Lapnurse through the blended learning method has been developed, with a face-to-face practical module and an online theoretical module. Findings obtained in this study regarding the successful design and preliminary validation by experts of the online module of the course suggest that Lapnurse could contribute to meet the training needs in MIS of Spanish and Portuguese nurses. In this way, they could be better able to work in the MIS field in their country or in another. Since the 1990s, changes in education policy, international relations, and the labor market facilitated the migration of nurses to work in other European countries. The recognition of qualifications under the OECD has facilitated such mobility of health workers [23]. In 2016, 145,487 nurses lived in a member state other than their country of citizenship [24]. Despite this high mobility rate, as far as we know, a standard curriculum of MIS nursing in Europe is still missing. Therefore, a new EU framework for nurses in MIS techniques should be developed. The content of courses such as Lapnurse should be applicable to European regulations so that such nurses trained in MIS could increase their employability in Europe and also contribute to the implantation of MIS at the European level.

Most studies about the validation of e-learning platforms are oriented to be performed by end users [17,25], but only few studies have been focused on instructors’ satisfaction [26,27]. The instructor’s point of view is often omitted but is also important [28], since the instructor is one of the main players of the learning process [29]. For that, in our work the validation tests have been performed by nurses with teaching experience in MIS.

One rather neglected issue in the validation tests is the reliability assessment of the e-learning environment. Due to its flexibility and ease of use, Moodle has been selected as the e-learning platform to provide the theoretical module of Lapnurse. It offers sufficient guarantees because it has been validated in several fields [30,31,32,33]. In this sense, system quality of an e-learning environment, in terms of navigability, accessibility, and stability [34,35], is critical for a good user experience of e-learning [36] and has a positive impact on use and satisfaction [37]. Therefore, we have successfully performed a functionality evaluation from a technical point of view, which takes into account the possible interaction between user, content, and technology.

The positive validation of contents highlights that they are whole and complete, updated, and correctly applied for educational purposes. This agrees with Back [38], who indicates that medical e-learning environments should be designed with availability of all relevant contents, always up-to-date, and with relevance for practical clinical work. Participants value contents as unique and innovative, but without reaching a score as high as the other questions. This feature should be improved in future editions of the course because one of the key webpage factors to obtain good SERP (Search Engine Ranking Positions) rating score from a search engine is the quality of contents, in the sense of being original and unique [39].

Some e-learning scenarios only provide unidirectional information for online use or downloads [38]. However, the learning outcomes and the engagement of users in their learning process can be favorably influenced by integrating interactivity [40], thus avoiding user dropouts in the e-learning environment. In Lapnurse, interactivity has been included through 3D designs of the glossary, which have been valued by participants as useful and complete, with interactive, realistic, and good quality 3D objects. The innovative 3D tools offered by this course provide originality and suppose a novelty compared with other e-learning scenarios. They would allow nurses to better know the instruments and equipment they have to manage during surgical interventions, since they could interact with them and not only see them in a simple picture or video as before in other online platforms.

Surgical videos and multimedia contents are important resources for the experts, agreeing thus with Bloomfield and Jones [41], which promotes video clips with clinical demonstrations as the most useful feature of e-learning due to being visual aids that can help students develop mental representations in their learning process. However, the incorporation of interactive components in surgical videos would be welcome, since interactive videos are being increasingly applied to skills-based training to overtake traditional passive watching of video-based training material [42].

User engagement with the e-learning platform can be achieved with usability features such as user-friendliness and ease of use [43]. Positive results from our usability validation show that the developed e-learning environment is attractive, organized, easy to use, and has a consistent design and layout with intuitive navigation. Despite this, ease of use has no influence on instructor satisfaction, but perceived usefulness and services quality on using the e-learning environment [29]. Participants have indicated that contents have good quality, provide added value and useful information, and are correctly applied for educational purposes. Moreover, they consider that the e-learning platform includes useful tools for users, such as the glossary of 3D designs, chat, and forums.

The Lapnurse course could be used as a basis for creating similar courses for training other surgical staff involved in laparoscopic interventions, such as surgeons or anesthetists. There are contents and sections related to laparoscopy in general that are common to any staff, such as ergonomics, instruments and equipment (including the glossary of interactive 3D designs), and diagnostic and approach techniques. It would only be necessary to adapt to the target staff some specific sections, such as laparoscopy for nursing. Moreover, this course could be generalized to other surgical disciplines for which the surgical staff need training, such as endoscopy, microsurgery, cardiovascular, etc. For that, taking into account the successful validation of usability and functionality of the e-learning platform, the structure and layout could be the same, and only the content of the course should be adapted to the surgical discipline. However, nowadays mobile internet and smartphones are increasingly popular in nursing education and practice [44], so structure and layout of the course can change from a PC to a mobile phone. According to Back [38], medical e-learning environments should have compatibility for all operating systems and unrestricted mobile access via tablet or smartphone.

There is a growing trend in the use of Massive Open Online Courses (MOOCs) in traditionally taught courses through this blended method [45]. But nevertheless, according to the definition of MOOC [46], maybe we cannot categorize our course as blended MOOC. First, a MOOC is massive, allowing the registration of thousands of participants. However, if we allow the registration of such a large number of participants in the online theoretical module of the course, it would be practically unfeasible to manage the practical module of the course, since this part is usually performed in specialized centres with capacity of 10–15 people each time. And second, a MOOC is open, in which any type of participants could register without any kind of restriction in prerequisite terms. However, Lapnurse is a specialization course of surgical staff, for which a minimum requirement of being a nurse is needed.

### 4.1. Study Limitations

More studies are needed to provide additional evidence from students at a national and international level performing a user-level validation, since only technicians and expert teachers have been involved in this preliminary validation.

### 4.2. Comparison with Prior Work

As far as we know, there are no similar blended learning laparoscopic courses specially addressed to nurses, so this original course with innovative 3D designs could improve their training in MIS as members of surgical teams involved in the interventions.

## 5. Conclusions

Although surgery is commonly present in training programs for nursing, Portuguese and Spanish nurses are inexperienced in MIS. Therefore, a laparoscopic training course for nursing has been developed through the blended learning method. The e-learning environment developed for the online theoretical module facilitates the accessibility to high quality didactic material to nurses in a remote and asynchronous way, allowing a greater implementation of the new MIS techniques in health systems.

Positive preliminary validation of Lapnurse could motivate developers to design similar courses. First, this kind of e-learning platform should preliminarily be validated to guarantee instructors’ satisfaction so that contents offered to final users can meet learning requirements. Second, contents should be complete, updated, and relevant for clinical practice and educational purposes, but also unique and innovative to attract users and thus stand out from the contents of similar e-learning platforms. Additionally, interactivity should be included in the platform, and even in surgical videos, to favor learning outcomes and to get users’ engagement. And, finally, reliability and quality of the system should be assessed for a good user experience. Such e-learning environments should be easy to use, attractive, and with intuitive navigation to favor users’ engagement, but with useful and quality services to meet the instructors’ satisfaction. Moreover, this kind of course should be developed for PC and mobile devices so that users can perform them anywhere, anytime, and on any device.

Successful preliminary validation of functionality, contents, and usability suggests that Lapnurse can solve the gap of training needs in MIS of both Spanish and Portuguese nurses. So, they could be properly qualified to work in this field in their own country, but also in the neighboring one. Since the obtained results were largely positive for valuing the e-learning environment and the blended learning method, it is a good starting point for considering in future studies the created course as a tool that could improve learning outcomes.

## Figures and Tables

**Table 1:** Results from the validation of specific contents of lessons 1–5 (Mean values ± Standard deviation).

Question	Lesson 1	Lesson 2	Lesson 3	Lesson 4	Lesson 5
	Mean ± SD	Mean ± SD	Mean ± SD	Mean ± SD	Mean ± SD
Q1	4.75 ± 0.50	4.75 ± 0.50	5.00 ± 0.00	5.00 ± 0.00	5.00 ± 0.00
Q2	5.00 ± 0.00	5.00 ± 0.00	5.00 ± 0.00	4.75 ± 0.50	4.75 ± 0.50
Q3	5.00 ± 0.00	4.75 ± 0.50	4.50 ± 0.58	5.00 ± 0.00	4.50 ± 0.58
Q4	4.75 ± 0.50	5.00 ± 0.00	5.00 ± 0.00	5.00 ± 0.00	5.00 ± 0.00
Q5	4.25 ± 0.50	4.75 ± 0.50	4.00 ± 1.41	4.75 ± 0.50	4.75 ± 0.50

**Table 2:** Results from the validation of specific contents of lessons 6–9 (Mean values ± Standard deviation).

Question	Lesson 6	Lesson 7	Lesson 8	Lesson 9
	Mean ± SD	Mean ± SD	Mean ± SD	Mean ± SD
Q1	4.75 ± 0.50	5.00 ± 0.00	5.00 ± 0.00	5.00 ± 0.00
Q2	4.75 ± 0.50	4.75 ± 0.50	4.50 ± 0.58	5.00 ± 0.00
Q3	4.25 ± 0.50	5.00 ± 0.00	5.00 ± 0.00	4.75 ± 0.50
Q4	5.00 ± 0.00	5.00 ± 0.00	5.00 ± 0.00	5.00 ± 0.00
Q5	4.75 ± 0.50	4.75 ± 0.50	4.75 ± 0.50	4.75 ± 0.50

## Data Availability

The data presented in this study are available on request from the corresponding author.

## Appendix A

This appendix includes the functional requirements evaluated:

- Access management requirements: user registration, login, logout, password recovery, and password change.
- Requirements of user profile and interactions: consultation and editing of personal data, user search, and language selection.
- Environmental maintenance requirements: user administration, monitoring, maintenance and updating of the environment, detection and notification of errors, and contingency response.
- Requirements for formal learning: creation, editing, and elimination of new courses, search for courses, request for subscription to courses, consultation of courses enrolled, accept/reject the subscription to a course, evaluation of skills acquired and progress evaluation.
- Requirements for informal learning: comment or raise questions in the forum, create new topics in the forum, and establish communication between users in the chat.

## Appendix B

This appendix includes the questionnaire for content validation.

Note: This questionnaire has been translated into English for the readers of this article. The original one that was used during the validation process was in Spanish and Portuguese.

### Questionnaire for Content Validation

Please read the different statements listed below concerning the specific content of each lesson of the theoretical module of the course and select the answer that best indicates the extent to which you agree with each of them, where 1 stands for “completely disagree” and 5 for “completely agree”.

- Q1: Contents have good technical quality
- Q2: The amount of content is appropriate
- Q3: The content distribution is appropriate
- Q4: Multimedia contents (images, videos, and 3D designs) are of quality, provide added value and useful information
- Q5: The questionnaire is adequate in terms of number of questions and difficulty

Please read the different statements listed below concerning the global content of the theoretical module of the course and select the answer that best indicates the extent to which you agree with each of them, where 1 stands for “completely disagree” and 5 for “completely agree”.

- Q6: The contents are unique, that is, the different types of content are not offered by similar e-learning environments
- Q7: The e-learning environment provides innovative content
- Q8: The e-learning environment uses professional language and appropriate content for users
- Q9: The content is whole and complete
- Q10: The e-learning environment uses multimedia content correctly applied for educational purposes
- Q11: Contents are updated
- Q12: Contents are easily understood
- Q13: 3D designs are interactive
- Q14: 3D designs are realistic and have good quality
- Q15: Contents are realistic and relevant to professional practice
- Q16: Surgical videos have good image quality
- Q17: The e-learning environment provides adequate means (questionnaires) to assess users’ knowledge on the learned topics
- Q18: The discussion forum of the e-learning environment is suitable and useful for users
- Q19: The e-learning environment provides a useful chat for communication between users
- Q20: Contents satisfactorily cover the theoretical aspects of a nursing laparoscopy course
- Q21: The glossary of 3D designs is useful and complete

Please answer the next two open questions:

- Q22: Indicate which lessons you consider more and less important
- Q23: Indicate which resources (glossary, chat, discussion forum, multimedia didactic content, lessons, questionnaires) you consider more and less important

## Appendix C

This appendix includes the questionnaire for usability validation.

Note: This questionnaire has been translated into English for the readers of this article. The original one that was used during the validation process was in Spanish and Portuguese.

### Questionnaire for Usability Validation

Please read the different statements concerning the usability of the e-learning environment listed below and indicate whether or not you agree with each of them

- Q24: The text-background color contrast is appropriate
- Q25: The font type and size facilitates reading
- Q26: Navigation labels are clear and concise
- Q27: The e-learning environment is attractive
- Q28: Appropriate number of buttons/links
- Q29: The e-learning environment uses colors properly
- Q30: The e-learning environment is organized
- Q31: The overall design of the e-learning environment is consistent (consistency and uniformity in style, structures and colors on all pages)
- Q32: Web pages within the e-learning environment are not long
- Q33: Navigation through the e-learning environment is intuitive
- Q34: Correct use is made of the visual space of the e-learning environment
- Q35: Information overload is avoided
- Q36: I like the graphic aspect and the general layout of the e-learning environment
- Q37: The e-learning environment is easy to use

## References

[1] Leone C., Conceição C., Dussault G. (2013). Trends of cross-border mobility of physicians and nurses between Portugal and Spain. Hum. Resour. Health.

[2] Sánchez-Peralta L.F., Sánchez-Fernández J., Pagador J.B., Sánchez-Margallo F.M. (2013). New technologies in minimally invasive surgery training: What do surgeons demand?. Cir. Cir..

[3] Graue M., Bjarkøy R.Ø., Iversen M.M., Haugstvedt A., Harris J. (2010). Integrating evidence-based practice into the diabetes nurse curriculum in Bergen. Eur. Diabetes Nurs..

[4] Kim K.K. (2010). Development of a web-based education program for nurses working in nursing homes on human rights of older adults. J. Korean Acad. Nurs..

[5] Öztürk D., Dinç L. (2014). Effect of web-based education on nursing students’ urinary catheterization knowledge and skills. Nurse Educ. Today.

[6] McVeigh H. (2009). Factors influencing the utilisation of e-learning in post-registration nursing students. Nurse Educ. Today.

[7] Ward J.A., Beaton R.D., Bruck A.M., de Castro A.B. (2011). Promoting occupational health nursing training: An educational outreach with a blended model of distance and traditional learning approaches. AAOHN J..

[8] Sowan A.K., Jenkins L.S. (2013). Designing, delivering and evaluating a distance learning nursing course responsive to students needs. Int. J. Med. Inf..

[9] Lahti M., Kontio R., Pitkänen A., Välimäki M. (2014). Knowledge transfer from an e-learning course to clinical practice. Nurse Educ. Today.

[10] Smyth S., Houghton C., Cooney A., Casey D. (2012). Students’ experiences of blended learning across a range of postgraduate programmes. Nurse Educ. Today.

[11] MOODLE. https://moodle.org/.

[12] Fernández-Alemán J.L., López-González L., González-Sequeros O., Jayne C., López-Jiménez J.J., Toval A. (2016). The evaluation of i-SIDRA–a tool for intelligent feedback–in a course on the anatomy of the locomotor system. Int. J. Med. Inf..

[13] Rapp A.K., Healy M.G., Charlton M.E., Keith J.N., Rosenbaum M.E., Kapadia M.R. (2016). YouTube is the most frequently used educational video source for surgical preparation. J. Surg. Educ..

[14] Sowan A.K., Idhail J.A. (2014). Evaluation of an interactive web-based nursing course with streaming videos for medication administration skills. Int. J. Med. Inf..

[15] Wallace T., Birch D.W. (2007). A needs-assessment study for continuing professional development in advances minimally invasive surgery. Am. J. Surg..

[16] Callisen L. Why Micro Learning Is the Future of Training in the Workplace. eLearning Industry. http://elearningindustry.com/micro-learning-future-of-training-workplace.

[17] Ortega-Morán J.F., Pagador J.B., Sánchez-Peralta L.F., Sánchez-González P., Noguera J., Burgos D., Gómez E.J., Sánchez-Margallo F.M. (2017). Validation of the three web quality dimensions of a minimally invasive surgery e-learning platform. Int. J. Med. Inf..

[18] De Góes Fdos S., Fonseca L.M., de Camargo R.A., de Oliveira G.F., Felipe H.R. (2015). Educational technology “Anatomy and Vital Signs”: Evaluation study of content, appearance and usability. Int. J. Med. Inf..

[19] Davids M.R., Chikte U., Grimmer-Somers K., Halperin M.L. (2014). Usability testing of a multimedia e-learning resource for electrolyte and acid-base disorders. Br. J. Educ. Technol..

[20] Guerrero-Martínez I.M., Portero-Prados F.J., Romero-González R.C., Romero-Castillo R., Pabón-Carrasco M., Ponce-Blandón J.A. (2020). Nursing Students’ Perception on the Effectiveness of Emergency Competence Learning through Simulation. Healthcare.

[21] Wu X.V., Chi Y., Selvam U.P., Devi M.K., Wang W., Chan Y.S., Wee F.C., Zhao S., Sehgal V., Ang N.K.E. (2020). A Clinical Teaching Blended Learning Program to Enhance Registered Nurse Preceptors’ Teaching Competencies: Pretest and Posttest Study. J. Med. Internet Res..

[22] Musharyanti L., Haryanti F., Claramita M. (2021). Improving Nursing Students’ Medication Safety Knowledge and Skills on Using the 4C/ID Learning Model. J. Multidiscip. Healthc..

[23] Galbany-Estragués P., Nelson S. (2018). Factors in the drop in the migration of Spanish-trained nurses: 1999–2007. J. Nurs. Manag..

[24] Fries-Tersch E., Tugran T., Bradley H. (2018). 2017 Annual Report on Intra-EULabour Mobility. European Commission. https://ec.europa.eu/futurium/en/system/files/ged/2017_report_on_intra-eu_labour_mobility.pdf.

[25] Ali M., Han S.C., Bilal H.S.M., Lee S., Kang M.J.Y., Kang B.H., Razzaq M.A., Amin M.B. (2018). iCBLS: An interactive case-based learning system for medical education. Int. J. Med. Inf..

[26] Swartz L.B., Cole M.T., Shelley D.J. (2010). Instructor satisfaction with teaching business law: Online vs. onground. Int. J. Inf. Commun. Technol. Educ. (IJICTE).

[27] Margalina V.M., De-Pablos-Heredero C., Botella J.L.M. (2018). Achieving Job Satisfaction for Instructors in E-Learning: The Relational Coordination Role. Social Issues in the Workplace: Breakthroughs in Research and Practice.

[28] Yengin I., Karahoca A., Karahoca D. (2011). E-learning success model for instructors’ satisfactions in perspective of interaction and usability outcomes. Procedia Comput. Sci..

[29] Almarashdeh I. (2016). Sharing instructors experience of learning management system: A technology perspective of user satisfaction in distance learning course. Comput. Hum. Behav..

[30] Costa C., Alvelos H., Teixeira L. (2012). The Use of Moodle e-learning Platform: A Study in a Portuguese University. Procedia Technol..

[31] Amandu G.M., Muliira J.K., Fronda D.C. (2013). Using Moodle E-learning Platform to Foster Student Self-directed Learning: Experiences with Utilization of the Software in Undergraduate Nursing Courses in a Middle Eastern University. Procedia Soc. Behav. Sci..

[32] Paragina F., Paragina S., Jipa A., Savu T., Dumitrescu A. (2011). The benefits of using MOODLE in teacher training in Romania. Procedia Soc. Behav. Sci..

[33] Escobar-Rodriguez T., Monge-Lozano P. (2012). The acceptance of Moodle technology by business administration students. Comput. Educ..

[34] Butzke M.A., Alberton A. (2017). Estilos de aprendizagem e jogos de empresa: A percepção discente sobre estratégia de ensino e ambiente de aprendizagem. REGE-Rev. Gestão.

[35] Tarhini A., Hone K., Liu X., Tarhini T. (2017). Examining the moderating effect of individual-level cultural values on users’ acceptance of e-learning in developing countries: A structural equation modeling of an extended technology acceptance model. Interact. Learn. Environ..

[36] Cidral W.A., Oliveira T., Di Felice M., Aparicio M. (2018). E-learning success determinants: Brazilian empirical study. Comput. Educ..

[37] Aparicio M., Bacao F., Oliveira T. (2017). Grit in the path to e-learning success. Comput. Hum. Behav..

[38] Back D.A., Behringer F., Haberstroh N., Ehlers J.P., Sostmann K., Peters H. (2016). Learning management system and e-learning tools: An experience of medical students’ usage and expectations. Int. J. Med. Educ..

[39] Rasheed K., Noman M., Imran M., Iqbal M., Khan Z.M., Abid M.M. (2018). Performance comparison among local and foreign universities websites using seo tools. ICTACT J. Soft Comput..

[40] Liaw S.Y., Wong L.F., Chan S.W., Ho J.T., Mordiffi S.Z., Ang S.B., Goh P.S., Ang E.N. (2015). Designing and evaluating an interactive multimedia Web-based simulation for developing nurses’ competencies in acute nursing care: Randomized controlled trial. J. Med. Internet Res..

[41] Bloomfield J.G., Jones A. (2013). Using e-learning to support clinical skills acquisition: Exploring the experiences and perceptions of graduate first-year pre-registration nursing students—A mixed method study. Nurse Educ. Today.

[42] Hammond J., Cherrett T., Waterson B. (2015). Making in-class skills training more effective: The scope for interactive videos to complement the delivery of practical pedestrian training. Br. J. Educ. Technol..

[43] Mai N., Heykyung P., Min-Jae L., Jian-Yuan S., Ji-Young O. (2015). Technology Acceptance of Healthcare E-Learning Modules: A Study of Korean and Malaysian Students’ Perceptions. TOJET Turk. Online J. Educ. Technol..

[44] Pimmer C., Brysiewicz P., Linxen S., Walters F., Chipps J., Gröhbiel U. (2014). Informal mobile learning in nurse education and practice in remote areas—A case study from rural South Africa. Nurse Educ. Today.

[45] Bralić A., Divjak B. (2018). Integrating MOOCs in traditionally taught courses: Achieving learning outcomes with blended learning. Int. J. Educ. Technol. High. Educ..

[46] Gonçalves B., Osório A. (2018). Massive Open Online Courses (MOOC) to improve teachers’ professional development. D-Rev. Educ. Distância Elearning.

